# Time-Since-Deposition Signatures for Canine Blood Based on Cellular Autofluorescence

**DOI:** 10.3390/vetsci12121183

**Published:** 2025-12-10

**Authors:** Alysia Townsley, Gabrielle Wolfe, Madison Smith, Arianna DeCorte, Amanda Elswick Gentry, Christopher J. Ehrhardt

**Affiliations:** Department of Forensic Science, Virginia Commonwealth University, Richmond, VA 23284, USA

**Keywords:** animal cruelty, autofluorescence, blood analysis, canine, flow cytometry, time since deposition, veterinary forensics

## Abstract

Animal cruelty investigations can be hampered by not knowing when a biological sample was deposited at a crime scene, i.e., the sample’s time-since-deposition (TSD). The goal of this study was to characterize a new signature system for estimating the TSD of canine blood samples based on changes in the autofluorescence spectra of cell populations that occur over time. The results showed that the intensity of cellular autofluorescence measured in two detector channels (523/30 nm and 695/50 nm; 488 nm excitation) could clearly differentiate older blood deposits (i.e., samples with a TSD of either 6 months or 1 year) from younger samples that had a TSD of less than three months. Further, cellular autofluorescence was strongly correlated with time when the sample TSD was between one day and three months, with correlation coefficients ranging between 0.83 and 0.91. A multiple linear regression model based on autofluorescence variation was tested on mock casework samples and yielded residual errors between 2 days and 35 days, with the lowest residuals observed in samples that have a TSD less than 6 days. This information can provide probative leads during an investigation of an illicit canine fighting event or an individual injury and also allow animal cruelty agencies to triage blood samples based on their TSD for downstream analyses.

## Simple Summary

The time-since-deposition (TSD) of bodily fluid stains can provide critical information during a forensic investigation of crimes involving animals by establishing whether biological evidence may have resulted from a suspected crime or if its deposition was unrelated. Although many techniques have been described for determining the TSD of human bodily fluids, few methods exist for the TSD of non-human samples. In this study, we describe a new method for estimating the TSD of animal bodily fluids, specifically, dried canine blood, that is based on changes over time in the intensity of autofluorescence from specific subpopulations of cells recovered from the sample. These changes are most drastic in samples that were aged for six months and one year. However, linear changes in autofluorescence intensity were also observed in samples with a TSD between one day and three months. A linear regression model was constructed from this data and used to estimate TSD in a series of blinded casework samples. Results indicate that this approach can potentially predict useful intervals of TSD for unknown canine blood samples, which can assist forensic investigators with crime reconstruction and identifying the most probative evidentiary samples for collection and analysis.

## 1. Introduction

Dog fighting is a pervasive form of organized animal cruelty, with thousands of dogs estimated to be involved in sport fighting in the U.S. alone and dozens of cases prosecuted each year across the U.S. [1]. The clandestine nature of fighting rings can also be a conduit for other types of organized crime, including gambling, narcotics, and illegal weapon trafficking [2,3]. This has led to the formation of a number of task forces within law enforcement agencies and partnerships that are dedicated to the investigation and prosecution of dog fighting and other types of animal crime [4,5]. One of the ongoing challenges in prosecuting animal fighting is the limited number of scientific techniques for analyzing physical evidence collected from the location of a suspected fight. While the canine CODIS DNA databank provides valuable information to law enforcement that may link an animal body fluid stain recovered from a crime scene to a specific animal and/or a suspect [6], key contextual information regarding how long ago the body fluid was deposited is often unknown [4]. Because of this, investigators may be uncertain whether the body fluid resulted from the fighting event or was deposited at a completely different time and was unrelated to the crime.

The analysis of animal body fluids and determining their time-since-deposition can also be relevant to other types of crimes. For example, canine bloodstains and/or bodily fluids have been used to link specific dogs to locations of physical abuse and/or neglect [4,7,8], as well as to associate person(s) with crime scenes or other human victims [9,10]. Animal abuse can also be a catalyst for larger federal investigations involving terrorism [11]. Regardless of the type of crime, biological samples from canines that are collected as evidence but have an unknown ‘time-since-deposition’ (TSD) can cause critical delays during the initial stages of the investigation and complicate the prosecution of the case, since the defendant can contest DNA findings by claiming that the presence of the fluid was unrelated to the crime in question. Questions about the timing of evidence deposition may also contribute to some animal welfare cases not being prosecuted at all [12].

Establishing the TSD of biological evidence has been an ongoing priority for the forensic community for many years [13,14] that has led to several methods being developed specifically for the analysis of blood stains. This includes spectroscopic signatures [15,16,17], colorimetric indicators [18], protein profiles [19,20], and genetic markers [21,22]. Another promising signature system for TSD is cellular autofluorescence profiles, which are created by the types and abundance of specific molecules within a sample that degrade over time after its deposition. Previous work has shown that autofluorescence variation over time may be used to reconstruct TSD for many human tissue types, including saliva [23], touch epidermal cells [24,25], and blood [26,27]. However, cellular autofluorescence profiling, like the other TSD signatures for blood listed above, has not been developed for any non-human tissue types.

Therefore, the goal of this study was to survey cellular autofluorescence variation in canine blood as a function of a sample’s TSD and build a framework for estimating TSD for unknown blood samples recovered as evidence. Because this is one of the first investigations of autofluorescence in canine bodily fluids, TSD signatures were initially investigated through a graphical exploration of the flow cytometry data and an empirical identification of a gating strategy to define subpopulations of cells that indicate TSD. This was followed by linear regression analysis that leveraged the appropriate subpopulations of cells to model the association between autofluorescence intensity and TSD. Lastly, this model was applied to a separate set of blinded canine blood samples to assess the accuracy of predicting TSD.

## 2. Materials and Methods

Canine blood samples used for this study were collected from small volumes (<1 mL) of unused blood that were left over from routine diagnostic tests (e.g., tests for heartworm) performed at veterinary clinics within Richmond, VA. All blood samples were anonymized without documentation of the breed, sex, or chronological age of the canine. The initial sample set used to characterize autofluorescence variation with TSD consisted of blood deposits from 21 different canines. A separate set of blood samples from 10 new canines was used for ‘blinded’ sample testing, i.e., samples where the TSD was unknown prior to analysis, which simulates a casework setting.

Dried blood samples representing different TSDs were created by dividing each canine blood sample into three 5 µL aliquots that were pipetted onto a glass slide and allowed to dry at room temperature (i.e., 21 °C–25 °C). Each slide was then stored under these conditions for the following periods of time (i.e., TSDs) for primary samples: 1 day, 3 days, 7 days, 14 days, 30 days, 60 days, 180 days, and 365 days. Mock casework samples were stored for TSDs: 2 days, 4 days, 6 days, 9 days, 13 days, 41 days, 65 days, 70 days, 71 days, and 146 days. At each designated TSD, dried blood deposits were collected from the glass slide with a cotton swab (Puritan Medical Products, Guilford, ME, USA; P/N 25-806 2WC) that was prewetted with filtered deionized water (0.2 µm filter, 18 MΩ) and then allowed to air dry. Each sample was stored under ambient conditions for its respective time period.

After incubation, each swab was placed upside down in a conical tube and incubated for 10 min in 1 mL of 1× PBS (Quality Biological, Gaithersburg, MD, USA; P/N 119-069-101). This was followed by vortex agitation for 30 s. This step released cells and other biological material from the swab into the solution. The swab was then discarded, and the resulting cell suspension in PBS buffer was immediately analyzed with flow cytometry.

The autofluorescence profiles of cell populations were collected using the Guava easyCyte^TM^ flow cytometer equipped with a 488 nm laser operating at 50 mW voltage (Cytek Biosciences, Fremont, CA, USA). Flow cytometry data for individual cells were analyzed with FlowJo v10 (Becton, Dickinson & Company, Franklin Lakes, NJ, USA). This included measurements on five different features: forward scatter (FSC), side scatter (SSC), and fluorescence intensity detected at three wavelengths, corresponding to the green channel (523/30 nm), the yellow channel (583/26 nm), and the red channel (695/50 nm). The number of cells detected in each sample across the entire time series varied between 34,488 cells and 197,705 cells. After data collection, autofluorescence profiles were saved in .xls format and exported to SPSS v31 (IBM, Inc., Chicago, IL, USA) for all quantitative analyses, which included the relative abundance/proportion of cell populations, Pearson’s correlation coefficient, multiple linear regression, and residual error analysis. Tabulated source data for autofluorescence profiles of blood cell populations is provided in the Supplementary Materials (Table S1).

## 3. Results

**Figure 1:**
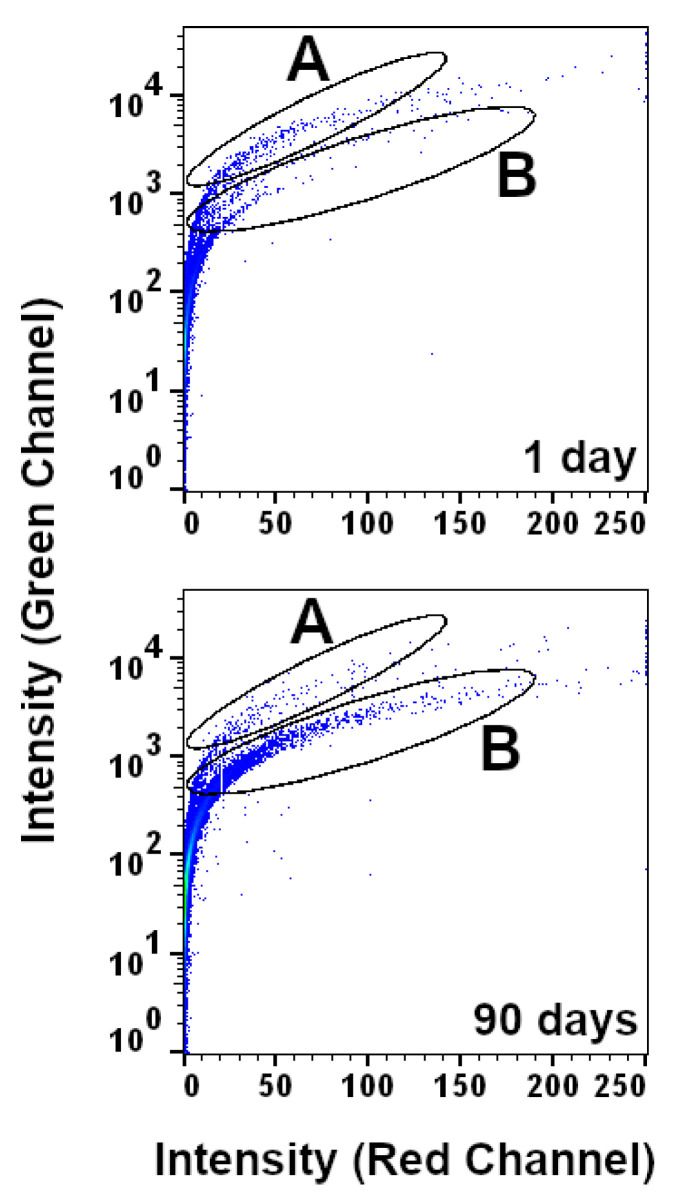
Dot density plot of two subpopulations of cells (A, B) used to characterize changes to blood samples with TSD. An example of changes in the relative abundance of cells across 1 day and 90 days in each panel.

**Figure 2:**
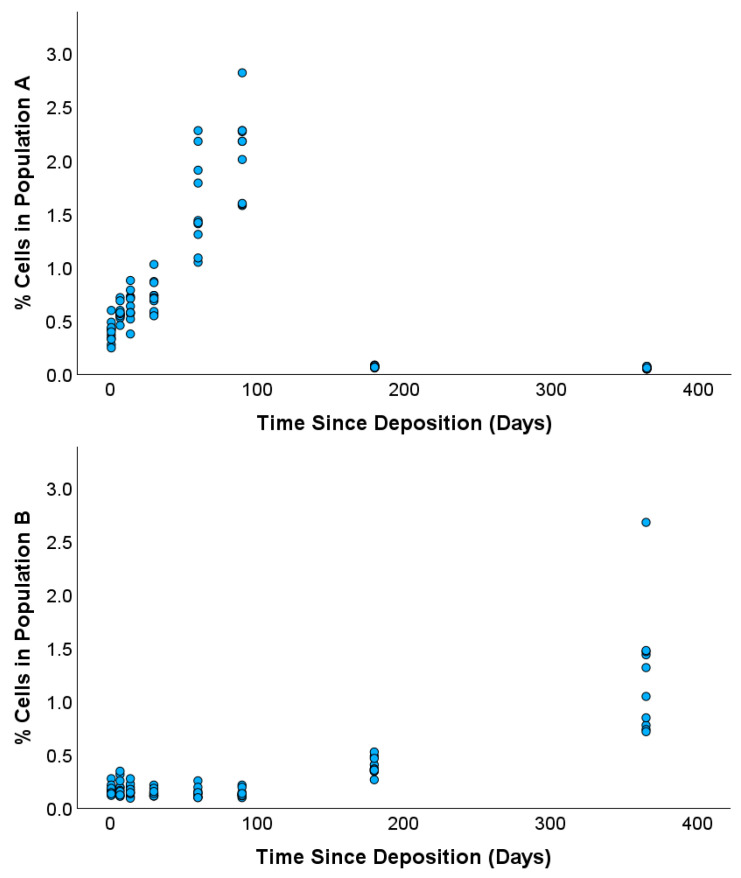
Relative abundance of cells within subpopulations A and B as TSDs vary between 1 day and ~1 year. Variation in subpopulation A is shown in the top panel, and variation in subpopulation B is shown in the bottom panel.

In an initial graphical survey of flow cytometry measurements, two subpopulations of cells were identified in plots of red autofluorescence intensity against green autofluorescence intensity that exhibited clear differences between samples with a TSD of 1 day and samples with a TSD of 90 days (Figure 1). Specifically, the proportion of cells falling into subpopulation ‘A’ decreased between the TSDs of 1 day and 90 days, while cells falling into subpopulation ‘B’ increased across the same time period. Based on this, the change in cell abundance for these two subpopulations was analyzed across the full TSD range between 1 day and 1 year. The results showed that the number of cells in population A increased from 0.2–0.6% (TSD 1 day) to 1.5–2.8% (TSD 90 days) and then decreased to 0.2–0.4% in samples with a TSD of ~180 days and 0–0.3% in samples with a TSD of 1 year (Figure 2A). Conversely, the relative abundance of cells in population B showed little variation between samples with TSDs ranging between 1 day and 90 days (ranging between 0.05% and 0.30%) and then increased to 0.3–0.5% in samples with a TSD of 180 days and 0.8–2.6% in samples with a TSD of 1 year (Figure 2B).

**Figure 3:**
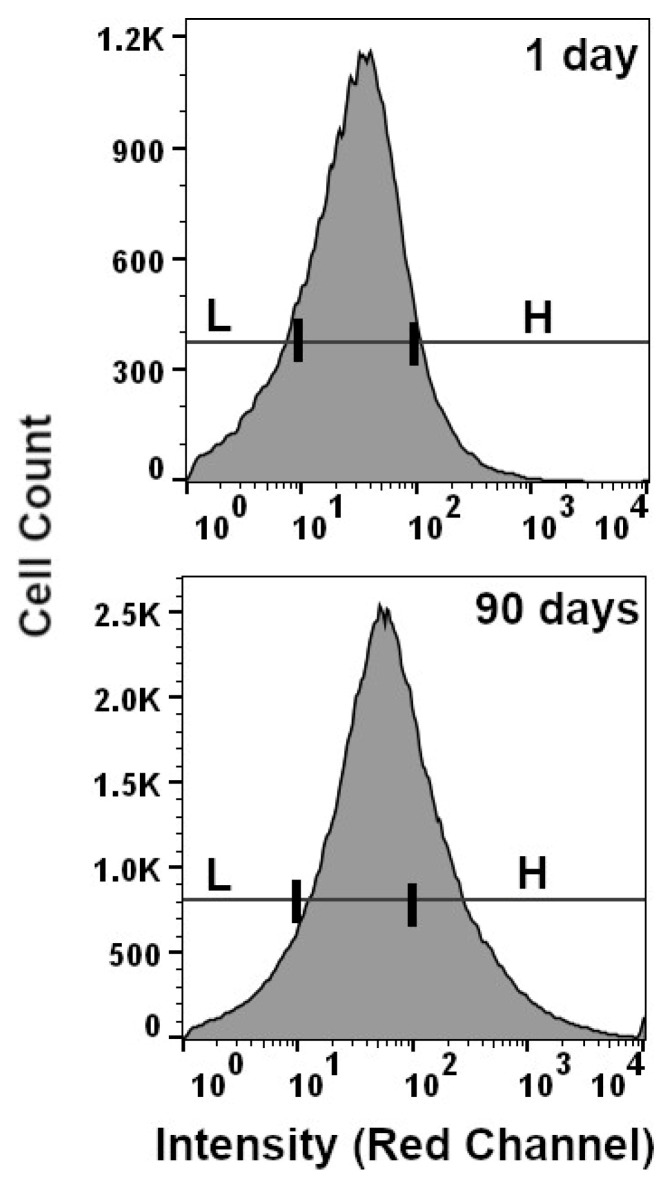
Low-fluorescence and high-fluorescence subpopulations of cells were used to model autofluorescence variation between TSDs of 1 day and 90 days. Low-fluorescence subpopulation in the red channel consisted of cells with autofluorescence intensity less than 10 RFU, and high-fluorescence subpopulations of cells consisted of cells with intensity greater than 100 RFU. Low and high threshold gates in both histograms are aligned vertically so autofluorescence shift with TSD is more easily visualized.

**Figure 4:**
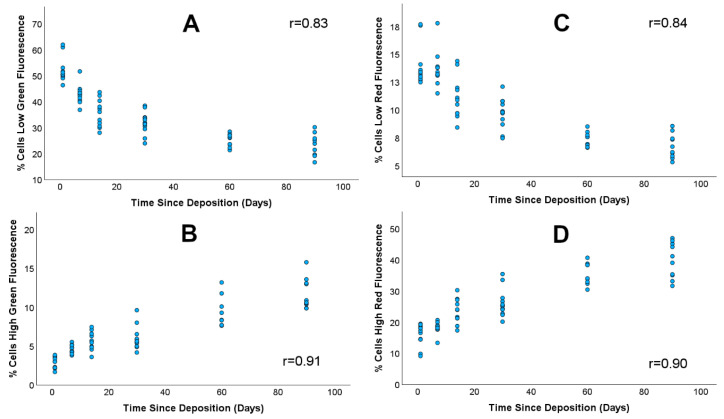
Variation in the proportion of cells within the low-fluorescence and high-fluorescence subpopulations with TSD. Low-fluorescence cells showed a decreasing abundance with TSD for the green and red channels (**A**,**C**), while high-fluorescence cells increased with TSD in the green and red channels (**B**,**D**).

Although the number of cells within these two subpopulations appears to differentiate samples with a TSD of either 6 months or 1 year from samples with a TSD less than 90 days, this signature did not show similar resolution between samples with a TSD that was 90 days or less (i.e., 1, 7, 14, 30, 60, or 90 days). Instead, clearer relationships between autofluorescence intensity time points between 1 day and 90 days were observed when each cell population is divided into four subpopulations comprising the proportion of cells with (1) intensity less than 10^2^ RFU in the green channel, (2) intensity greater than 10^3^ RFU in the green channel, (3) intensity less than 10^2^ RFU in the red channel, and (4) intensity greater than 10^3^ RFU in the red channel. An example of the gates that define these subpopulations is shown in Figure 3 for the red channel. When the proportion of cells in each of these subpopulations was plotted against TSD, both linear and nonlinear changes were observed. For example, the low-intensity-green fluorescence (less than 10^2^ RFU) subpopulation constituted ~ 55% of the total cell population in samples with a TSD of 1 day and then decreased linearly to ~ 20% of the cell population for samples with a TSD of 90 days (Figure 4C,D). Conversely, the proportion of cells with high-intensity green fluorescence (greater than 10^3^ RFU) was only 3–6% for samples with a TSD of 1 day and then increased steadily to 10–15% for samples with a TSD of 90 days, followed by an increase to ~50–60% for samples with a TSD of 1 year (Figure 4A,B).

Subpopulations within the red fluorescence channel showed similar trends, with the low-intensity subpopulation comprising ~11% to ~14% of the total cell population in samples with a TSD of 1 day and decreasing to 5–8% for samples with a TSD of 90 days (Figure 4A,C). Cells within the high-red fluorescence subpopulation started with a range of 10–20% for 1-day TSD samples and then increased to 30–50% in samples with a TSD of 90 days (Figure 4B,D). The abundance of cells in each of the four subpopulations also showed high-magnitude, linear correlations with TSD, with Pearson’s correlation coefficients of 0.83 and 0.84 for low-fluorescence green and red subpopulations and 0.91 and 0.90 for high-fluorescence red and green subpopulations (Figure 4).

Based on the correlations between cell subpopulation autofluorescence and time, a multiple linear regression model was built to predict the TSD of unknown samples using the proportion of cells within the low-green, high-green, low-red, and high-red subpopulations as the predictor variables. The resulting regression model showed an explained variance (i.e., adjusted R^2^) of 0.84 and a standard error of the estimate (SEE) of ~12.2 days. When this model was tested on an independent set of blinded, mock casework samples, the predicted TSD values using the regression model were generally consistent with actual TSDs, particularly for samples with a TSD of 6 days or less (Table 1). For example, of the nine blood samples with a true TSD of 6 days or less, the difference between the predicted TSD and actual TSD ranged between 1 day and 7 days, with an average difference of ~3 days. The difference between predicted and actual TSD increased in samples with older TSDs; e.g., samples with a TSD greater than 6 days showed an average residual of ~17 days (ranging between 5 days and 35 days; Table 1).

## 4. Discussion

Overall, results from this proof-of-concept study show two novel signatures for the TSD of canine blood that are based on the abundance of cells falling into discrete subpopulations defined by the intensity of autofluorescence across the green and red emission channels. One set of subpopulations can be used to easily distinguish blood samples with TSDs of 6 months or 1 year from samples that have a TSD that is less than 90 days (i.e., the ratio of cells in the ‘A’ and ‘B’ groups shown in Figure 1). The second set of subpopulations is defined by the proportion of cells falling into ‘low’ or ‘high’ fluorescence and showed more linear changes with increasing TSD between 1 day and 90 days. Modeling autofluorescence variation from the latter subpopulation using linear regression indicated that TSD may be inferred in blinded/mock casework samples with residual errors ranging between ~3 days and ~17 days, depending on the actual TSD of the sample. Direct comparison of these results to previous TSD research is challenging, given the paucity of studies on canine blood specifically and the fact that most foundational research on human blood TSD encompasses a wide range of sampling conditions, time intervals, methodological approaches, and metrics for assessing error associated with TSD prediction. However, the observed changes in the cellular autofluorescence of canine blood are consistent with fluorescence-based cellular changes with TSD that have been described for human bloodstains. For example, both linear and nonlinear changes in fluorescence signatures were observed in human bloodstains that were aged for up to 90 days [26,27]. Similarly, signatures for TSD based on completely orthogonal approaches have generally reported high correlations between the respective biological metrics and TSD, i.e., correlation coefficients greater than 0.8 for colorimetric [18], spectroscopic [28], and RNA-based signatures [29], which are largely consistent with the correlations between cellular autofluorescence and TSD observed in this study.

As with most proof-of-concept work, adding samples to the regression model, particularly those with TSDs not represented in the current dataset, will likely improve the precision of TSD estimates of canine blood deposits. Additionally, characterizing the effect that environmental factors and alternate substrates have on autofluorescence variation is needed to understand the types of investigative settings where this type of analysis can be used. Variables such as temperature, humidity, exposure to sunlight, and the porosity of the sample substrate have been shown to affect biochemical signatures for a variety of human biological sample types [30,31,32] and may similarly impact the forensic signatures associated with canine blood stains. Given the wide range of depositional environments in which animal body fluids may be found (e.g., indoor residences, outdoor facilities) as well as substrates from which biological stains may be collected, incorporating these factors into a TSD signature model will increase its utility for animal cruelty investigations.

Nevertheless, the autofluorescence signatures described here may have immediate applications for presumptive determinations of TSD for unknown blood stains within certain windows of time, e.g., whether blood stains were deposited within the last week or blood stains are several months old. This information can provide investigative leads as well as allow animal cruelty agencies to triage samples from complex crime scenes based on TSD, potentially saving time and resources when processing samples for DNA profiling or other downstream analyses.

We anticipate that this work will add to the collection of forensic techniques that were originally developed for human biological evidence and adapted for the analysis of animal body fluids within the field of veterinary forensics [33,34]. Although the number of techniques is growing [35], many animal welfare agencies may have limited resources to execute the full range of forensic laboratory tests, especially when investigations may involve large crime scenes and dozens of animal victims (e.g., Michael Vick Investigation [36]). Towards this end, we have optimized a method for TSD determination that requires minimal laboratory reagents and consumables (e.g., PBS buffer, microcentrifuge tubes, swabs) and can also be performed in a rapid and high-throughput manner—usually less than 5 min per sample. Further, the interpretation of cellular autofluorescence signatures for TSD does not require complex algorithms or probabilistic frameworks. Instead, TSD estimates can be made using either a nearly binary autofluorescence threshold (for samples that are 6 months or older) or a linear regression model (for samples 90 days or younger). Lastly, flow cytometry itself is a widely accepted method for a variety of biomedical and clinical applications [37,38], with instrumentation available at numerous academic and industrial laboratory facilities, which may facilitate its transition to casework. Continuing to expand the scientific capabilities for agencies tasked with investigating animal cruelty in this way will inevitably yield more probative information from physical evidence, and, ultimately, better judicial outcomes.

## Figures and Tables

**Table 1:** Predicted TSD for blinded canine blood samples.

Blind Sample #	Actual TSD (Days)	Estimated TSD (Days)	Difference (Residual)
**1**	2	<1	2
**2**	2	<1	2
**3**	2	<1	2
**4**	4	<1	4
**5**	4	6.3	2
**6**	4	<1	4
**7**	6	<1	6
**8**	6	12.9	7
**9**	6	5.0	1
**10**	9	19.1	10
**11**	9	7.0	2
**12**	9	14.0	5
**13**	13	10.0	3
**14**	13	22.6	9
**15**	13	23.2	10
**16**	41	32.6	9
**17**	41	16.1	25
**18**	41	31.2	10
**19**	65	56.5	9
**20**	65	80.1	15
**21**	65	44.0	21
**22**	71	58.4	13
**23**	71	36.9	35
**24**	71	64.4	7
**25**	70	64.7	5
**26**	146	181.7	35

## Data Availability

The original contributions presented in this study are included in the article/Supplementary Materials. Further inquiries can be directed to the corresponding author.

## Supplementary Materials

The following supporting information can be downloaded at: https://www.mdpi.com/article/10.3390/vetsci12121183/s1, Table S1: Tabulated Source Data for Cell Population Autofluorescence Plots in Figure 2 and Figure 4.
